# Translation and cross-cultural adaptation of the International Trauma Questionnaire for use in Brazilian Portuguese

**DOI:** 10.1590/1516-3180.2019.0066070519

**Published:** 2019-08-29

**Authors:** Júlia Candia Donat, Nathália dos Santos Lobo, Gabriela dos Santos Jacobsen, Eduardo Reuwsaat Guimarães, Christian Haag Kristensen, William Berger, Mauro Vitor Mendlowicz, Eduardo de Paula Lima, Alina Gomide Vasconcelos, Elizabeth Nascimento

**Affiliations:** I MSc. Psychologist and Researcher, Postgraduate Program on Psychology, Pontifícia Universidade Católica do Rio Grande do Sul (PUCRS), Porto Alegre (RS), Brazil.; II Psychology Undergraduate Student, Pontifícia Universidade Católica do Rio Grande do Sul (PUCRS), Porto Alegre (RS), Brazil.; III BSc. Psychologist and Researcher, Postgraduate Program on Psychology, Pontifícia Universidade Católica do Rio Grande do Sul (PUCRS), Porto Alegre (RS), Brazil.; IV MSc. Psychologist and Researcher, Postgraduate Program on Psychology, Pontifícia Universidade Católica do Rio Grande do Sul (PUCRS), Porto Alegre (RS), Brazil.; V PhD. Psychologist and Professor, Postgraduate Program on Psychology, Pontifícia Universidade Católica do Rio Grande do Sul (PUCRS), Porto Alegre (RS), Brazil.; VI PhD. Psychiatrist and Professor, Institute of Psychiatry, Universidade Federal do Rio de Janeiro (UFRJ), Rio de Janeiro (RJ), Brazil.; VII PhD. Psychiatrist and Professor, Institute of Psychiatry, Universidade Federal do Rio de Janeiro (UFRJ), and Department of Psychiatry and Mental Health, Universidade Federal Fluminense (UFF), Rio de Janeiro (RJ), Brazil.; VIII PhD. Psychologist and Postdoctoral Student, Department of Psychology, Universidade Federal de Minas Gerais (UFMG), Belo Horizonte (MG), Brazil.; IX PhD. Psychologist and Postdoctoral Student, Department of Psychology, Universidade Federal de Minas Gerais (UFMG), Belo Horizonte (MG), Brazil.; X PhD. Psychologist and Professor, Department of Psychology, Universidade Federal de Minas Gerais (UFMG), Belo Horizonte (MG), Brazil.

**Keywords:** Trauma and stressor related disorders, Psychological trauma, Psychiatry, International classification of diseases, Surveys and questionnaires

## Abstract

**BACKGROUND:**

The most recent editions of diagnostic manuals have proposed important modifications in posttraumatic stress disorder (PTSD) criteria. The International Trauma Questionnaire (ITQ) is the gold-standard measurement for assessing PTSD and complex PTSD in accordance with the model of the 11^th^ International Classification of Diseases (ICD-11).

**OBJECTIVE:**

The aim of this study was to adapt the ITQ for the Brazilian context.

**DESIGN AND SETTING:**

The translation and cross-cultural adaptation of the ITQ for use in Brazilian Portuguese was performed in trauma research facilities in Porto Alegre, Rio de Janeiro and Belo Horizonte, Brazil.

**METHODS:**

The adaptation followed five steps: (1) translation; (2) committee synthesis; (3) experts’ evaluation through the content validity index (CVI) and assessment of interrater agreement though kappa statistics; (4) comprehension test with clinical and community samples (n = 35); and (5) final back-translation and authors’ evaluation.

**RESULTS:**

Two independent translations were conducted. While working on a synthesis of these translations, the committee proposed changes in six items to adapt idiomatic expressions or to achieve a more accurate technical fit. Both the expert judges’ evaluation (CVI > 0.7; k > 0.55) and the pretest in the target population (mean comprehension > 3) indicated that the adapted items were adequate and comprehensible. The final back-translation was approved by the authors of the original instrument.

**CONCLUSION:**

ITQ in its Brazilian Portuguese version achieved satisfactory content validity, thus providing a tool for Brazilian research based on PTSD models of the ICD-11.

## INTRODUCTION

Posttraumatic stress disorder (PTSD) has high prevalence worldwide,^1^ and it is frequently diagnosed by mental health professionals.^2^^,^^3^ However, controversies surround this diagnosis. The high number of symptoms, among which some are present in other mental disorders (e.g. detachment from others, sleep disturbance, concentration problems and reckless behavior), leads to high rates of comorbidities.^4^^,^^5^^,^^6^ Furthermore, there are studies investigating a certain type of PTSD that is different from what is described in diagnostic manuals. When repeated exposure to trauma is associated with symptoms such as emotional dysregulation, dissociation and negative self-concept, the reaction is often described as “disorder of extreme stress not otherwise specified” or complex PTSD.^7^^,^^8^ In the literature on trauma, PTSD criteria are often discussed, particularly with regard to which general symptoms of psychological distress should be understood as frequent comorbidities and not as part of the disorder; and which responses are directly related to trauma and therefore should be added to the diagnosis.^8^^,^^9^^,^^10^

The most recent version of the International Classification of Diseases (ICD-11) sought to encompass current scientific knowledge and proposed a new model for PTSD, in which the basic and complex forms of PTSD were distinguished and many symptoms that were considered to relate to general distress were eliminated. PTSD is described as a reaction to trauma that includes (1) re-experience of the traumatic event (i.e. vivid intrusive memories, flashbacks or nightmares accompanied by overwhelming emotions); (2) avoidance of thoughts, memories, situations, people or activities reminiscent of the event; and (3) a state of perceived current threat in the form of hypervigilance or enhanced startle reactions to stimuli such as unexpected noises. Complex PTSD is described as a disorder that typically arises after an extreme or prolonged stressor from which escape is difficult or impossible (e.g. childhood sexual abuse, torture or prolonged domestic violence) and would comprise the sum of PTSD and more persistent symptoms of disturbances in self-organization (DSO), in three clusters: (1) affective dysregulation (e.g. self-destructive behavior, emotional anesthesia or dissociative states); (2) persistent beliefs about oneself as diminished, defeated or worthless, accompanied by feelings of shame, guilt or failure; and (3) persistent difficulties in maintaining relationships and feeling close to others. Both diagnoses require that symptoms cause significant impairment in important areas of functioning, such as social, educational or occupational.^11^^,^^12^

Another important diagnostic guide, the 5^th^ edition of the Diagnostic and Statistical Manual of Mental Disorders (DSM-5), provides a broader approach towards defining this phenomenon. Instead of separating posttraumatic reactions into two conditions and reducing the overall number of symptoms (as in ICD-11), this approach does not eliminate any previous criteria and adds symptoms that are often associated with repeated exposure to trauma, to make a unique diagnosis through a new cluster of negative alterations in cognition and mood (e.g. overly negative thoughts and assumptions about oneself or the world, or difficulty in experiencing positive affect) and a new dissociative subtype*.*^13^

Differences in the definition of posttraumatic symptoms may impact the work of clinicians and researchers in an important way. Current diagnostic differences should be investigated through empirical work, with the aim of evaluating the validity of both models in different cultures and populations, in order to achieve overall comprehension of posttraumatic reactions and to define approaches for future editions of diagnostic manuals.^14^^,^^15^ Thus, it is necessary that instruments representing these new models are available in as many languages as possible. In Brazil, an adaptation of the PTSD model of the DSM-5 has already been produced,^16^ but this has not yet been done for the model of the ICD-11.

The International Trauma Questionnaire (ITQ) is the gold-standard tool for evaluating the ICD-11 model of PTSD and complex PTSD. The ITQ is a 12-item questionnaire, with evidence of factorial, discriminant and concurrent validity in its original version.^17^ This instrument has already been used to investigate the ICD-11 model of PTSD and complex PTSD in different countries such as Australia,^18^ Germany, Lithuania, United States, United Kingdom,^19^ West Papua,^20^ Lebanon^21^ and Uganda.^22^ To our knowledge, no similar studies have yet been conducted in Latin America. It is especially relevant to have measurements for investigating posttraumatic reactions in Brazil, since this country presents high rates of exposure to traumatic events such as urban violence and traffic accidents.^23^^,^^24^ Many instruments used in Brazilian settings were originally designed in other languages. Adaptation of instruments is an important step in ensuring the quality of these measurements, with the aim of maintaining content validity (i.e. the extent to which a measurement represents the construct) while still addressing important cultural and linguistic factors of relevance to the target population.^25^^,^^26^

## OBJECTIVE

The aim of this study was to translate and culturally adapt the ITQ for use in Brazilian Portuguese, which will enable adequate investigation of the PTSD and complex PTSD models of the ICD-11 in Brazil.

## METHOD

The ITQ is a gold-standard open-access tool for investigating PTSD and complex PTSD as defined by the ICD-11. In the present study, the ITQ in its final 12-item version, which was recently finalized after extensive empirical work, was used.^17^ The instrument comprises two diagnostic questionnaires: one for PTSD and the other for DSO. The responses in both of these questionnaires are measured on five-point Likert scales ranging from 0 (not at all) to 4 (extremely) with six items divided into three clusters (for PTSD, re-experience, avoidance and state of perceived current threat; for DSO, affective dysregulation, negative self-concept and disturbances in relationships); plus three items for identifying functional impairment. For the diagnosis of PTSD, it is necessary to score at least one symptom in each cluster > 2 (on the Likert scale) with a functional impairment score also > 2 in at least one item, in the PTSD questionnaire. For complex PTSD, it is necessary to score at least one symptom > 2 in each cluster in both the PTSD and the DSO questionnaire, also with a functional impairment score > 2 in at least one item in both questionnaires.

The cross-cultural adaptation was based on the guidelines of the International Test Commission,^25^ on data in the specialized literature^26^^,^^27^^,^^28^^,^^29^^,^^30^ and on adaptations of posttraumatic measurements in previous studies.^16^^,^^31^^,^^32^ It followed five steps: (1) translation; (2) committee synthesis; (3) expert judges’ evaluation through the content validity index (CVI) and assessment of interrater agreement though kappa coefficients; (4) comprehension test with clinical and community samples (n = 35); and (5) final back-translation and authors’ evaluation.

1. Translation - The original version of ITQ was translated into Brazilian Portuguese by two bilingual Brazilian psychologists (both with MSc degrees) with expertise in trauma-related disorders. Independently, each translator created one translated version of the instrument.
2. Committee synthesis - A committee of academics compared the two translated versions with the original ITQ to certify that all items expressed the same ideas, in order to achieve semantic, idiomatic and conceptual equivalences. The committee was composed of undergraduate, master’s and doctoral students. Items were chosen from either of the translated versions and, whenever necessary, were changed and refined by the committee. This step generated a unified version of the ITQ in Brazilian Portuguese.
3. Expert judges’ evaluation *-* The unified version was evaluated through the content validity index (CVI), which is used to quantify the adequacy of items within a certain context. The quality of the items should be judged by a group of experts within the construct that the instrument is supposed to measure. The CVI indicates the clarity, coherence and semantic correspondence of the scale, in relation to the original version. In our study, CVI responses were given independently by three judges in different Brazilian cities, in order to minimize the impact of regional speech patterns: one psychologist (MSc) who was an expert in trauma-related disorders, in Porto Alegre; one psychiatrist (PhD) who was an expert in trauma-related disorders, in Rio de Janeiro; and one psychologist (PhD) who was an expert in cross-cultural adaptation of instruments, in Belo Horizonte. The judges had to evaluate the relevance of each item through a five-point Likert scale, ranging from 0 (“not at all”) to 4 (“totally”), in three dimensions: (1) language clarity, which measures how comprehensible the items are; (2) practical relevance, which measures how adequate each item is to the target population; and (3) theoretical relevance, which measures how much the item agrees with the construct theory. Following this, CVI scores were calculated in accordance with the specific CVI formula, based on division of the mean values given by the experts (for further details, see Cassepp-Borges, Balbinotti & Teodoro, 2010^27^). Items with CVI lower than 0.7 would be rephrased and repeatedly resubmitted to the three judges until a satisfactory value was reached.^25^ Also, the judges were asked to indicate to which questionnaire (i.e. PTSD or DSO) each item belonged. The degree of agreement between the judges would be assessed through Cohen’s weighted kappa. In accordance with the guidelines,^33^ kappa scores > 0.41 would indicate moderate agreement, > 0.61 substantial agreement and > 0.81 almost perfect agreement.
4. Comprehension test - Thirty-five individuals in three Brazilian state capitals (Belo Horizonte, Rio de Janeiro and Porto Alegre) were independently asked about the ease of comprehension of all items of the pre-final version, with responses on a five-point Likert scale ranging from 1 (“I didn’t understand anything”) to 5 (“I completely understood”). The indicators of understanding were the central trend scores (mean) and dispersion (standard deviation) for each item. Sample size and satisfactory understanding (mean score > 3) were defined based on previous studies.^16^^,^^29^^,^^30^ A convenience sample was recruited from research facilities in all three cities. Almost half of the sample (45.7%; n = 16) was composed of trauma victims seeking treatment at psychological or psychiatric centers at the universities that collaborated in this study; and the remaining sample was composed of individuals within the general population (54.3%; n = 19) who were mostly friends and family of the clinical population that participated in the study. The goal was to recruit a sample of participants with different sociodemographic characteristics and backgrounds, to increase the validity of the instrument. The sample was heterogeneous in terms of gender distribution (37.1% men [n = 13] and 62.9% women [n = 22]); educational level (17.1% with primary education [n = 6], 31.4% with secondary education [n = 11] and 51.5% with higher education [n = 18]); and state of residence (28.6% in Rio Grande do Sul [n = 10], 31.4% in Minas Gerais [n = 11] and 40% in Rio de Janeiro [n = 14]).
5. Backtranslation *-* Lastly, the final Brazilian Portuguese version was back-translated by a bilingual translator, with a major in Languages, with no expertise in mental health and blinded to the original instrument. The backtranslation result was sent to the authors of the original study for their final approval.

This study was approved by the Ethics Committee of the Pontifícia Universidade Católica do Rio Grande do Sul (approval number 2.558.869; March 2018). All participants were informed about the objectives of this study and regarding its voluntary nature, with confidentiality assured, and they signed an informed consent form before filling out the questionnaires. This study was conducted between March and August 2018.

## RESULTS

In making the synthesis from the two translated versions, the committee proposed minor changes regarding the use of idiomatic expressions or with the aim of achieving a more accurate technical fit in four items of the PTSD questionnaire (items 2, 3, 4 and 5) and in two items of the DSO questionnaire (items 1 and 5).

1. In item 2 of the PTSD questionnaire, the term “intensas” (= intense) was chosen over “poderosas” (the literal translation of “powerful”, which had been chosen by both translators) to describe trauma memories, because the term “poderosas” has a positive connotation in Brazilian Portuguese.
2. The best translation for the word “reminders”, present in items 3 and 4 of the PTSD questionnaire, was extensively discussed. Both translators suggested terms that could be related to memories (“lembranças” and “lembretes”). This was considered by the committee to be inadequate because of item 4, which refers to external reminders of the trauma. The term “pistas” (= ­triggers or cues) was chosen.
3. Item 5 of the PTSD questionnaire contained the term “super-alert” to describe hyperarousal symptoms. Both translators chose the literal term “super-alertas”, but the committee decided that “hiperalertas” (= hyper-alert) would be a better technical fit for this expression in Brazilian Portuguese.
4. In item 1 of the DSO questionnaire, the term “upset” was translated as “triste” (= sad) or “abalado” (= shaken). Neither of these was considered appropriate by the committee, who defined the word “chateado” (another possible translation of “upset”) as more adequate.
5. In item 2 of the DSO questionnaire, the expression “cut off” was translated as “afastado” (= away) or “isolado” (= isolated), but was rewritten as “desconectado” (= detached) by the committee.

The unified version created through the committee’s synthesis was evaluated by the three judges in different Brazilian districts using the CVI. The results from the expert judges’ evaluation showed that all items were considered adequate (> 0.7), as seen in Table 1. One change was made in response to the experts’ comments and suggestions, in order to achieve a more appropriate expression. In item 1 of the DSO questionnaire, the term “demoro bastante tempo” replaced “levo bastante tempo” to translate “takes me a long time”. Cohen’s weighted kappa coefficient indicated overall agreement of 77.8% between the judges, with PTSD items showing k = 0.56 [95% confidence interval, CI, 0.18-0.93] and DSO items showing k = 0.55 [95% CI, 0.22-0.88], thus indicating moderate agreement between the judges with regard to deciding which questionnaire each item belonged to.

**Table 1.** Expert judges’ evaluation, pretesting in the target population and backtranslation of the Brazilian Portuguese Version of the International Trauma Questionnaire (ITQ)

	Original version	Brazilian version	CVI- LC	CVI- PR	CVI-TR	Population evaluation (mean [SD])	Backtranslation
PTSD questionnaire							
1	Having upsetting dreams that replay part of the experience or are clearly related to the experience?	Ter sonhos desagradáveis que reproduzem parte da experiência ou são claramente relacionados à experiência?	0.82	0.96	0.96	4.26 [1.20]	Having unpleasant dreams that reproduce part of the experience or are clearly related to it?
2	Having powerful images or memories that sometimes come into your mind in which you feel the experience is happening again in the here and now?	Ter imagens ou memórias intensas que, às vezes, vêm a sua mente, fazendo com que você sinta que a experiência está acontecendo novamente no aqui e agora?	0.96	0.96	0.96	4.38 [1.02]	Having intense images or memories that sometimes come to your mind, making you feel that the experience is happening again, here and now?
3	Avoiding internal reminders of the experience (for example, thoughts, feelings, or physical sensations)?	Evitar pistas internas da experiência (por exemplo, pensamentos, sentimentos ou sensações físicas)?	0.76	0.96	0.96	3.71 [1.43]	Avoiding internal cues of the experience (for instance, thoughts, feelings or physical sensations)?
4	Avoiding external reminders of the experience (for example, people, places, conversations, objects, activities, or situations)?	Evitar pistas externas da experiência (por exemplo, pessoas, lugares, conversas, objetos, atividades ou situações)?	0.96	0.96	0.96	4.29 [1.27]	Avoiding external cues of the experience (for instance, people, places, conversations, objects, activities or situations)?
5	Being “super-alert”, watchful, or on guard?	Estar “hiper-alerta”, vigilante ou em guarda?	0.89	0.96	0.96	4.74 [0.56]	Being hyper-alert, vigilant or on guard?
6	Feeling jumpy or easily startled?	Sentir-se sobressaltado(a) ou facilmente assustado(a)?	0.82	0.96	0.89	4.54 [0.89]	Feeling startled or easily scared?
DSO questionnaire							
1	When I am upset, it takes me a long time to calm down	Quando estou chateado(a), demoro bastante tempo para me acalmar	0.89	0.96	0.96	4.86 [0.35]	When I’m upset, it takes quite a long time to calm me down again
2	I feel numb or emotionally shut down.	Eu me sinto anestesiado(a) ou emocionalmente desligado(a)	0.76	0.96	0.96	4.57 [0.85]	I feel insensitive and emotionally disconnected
3	I feel like a failure	Eu me sinto um fracasso	0.96	0.89	0.96	4.83 [0.38]	I feel like a failure
4	I feel worthless	Eu me sinto sem valor	0.96	0.96	0.96	4.74 [0.74]	I feel worthless
5	I feel distant or cut off from people.	Eu me sinto distante ou desconectado(a) de outras pessoas	0.96	0.96	0.96	4.74 [0.74]	I feel distant or disconnected from other people
6	I find it hard to stay emotionally close to people.	Acho difícil ficar emocionalmente próximo(a) de outras pessoas	0.89	0.96	0.96	4.71 [0.62]	It’s difficult for me to be emotionally attached to other people

CVI-LC = Content Validity Index - Language Clarity; CVI-PR = Content Validity Index - Practical Relevance; CVI-TR = Content Validity Index - Theoretical Relevance; SD = standard deviation; PTSD = Posttraumatic Stress Disorder; DSO = Disturbances in Self-Organization.

Table 1 also shows the results from the comprehension test on the target population. All items were considered comprehensible (> 3) in the first round of evaluations. The backtranslation result was compared with the original version and was approved by the authors of the original study. Appe ndix 1 presents the final version of the Brazilian version of the ITQ.

## DISCUSSION

This study reports on the cross-cultural adaptation of ITQ for use in Brazilian Portuguese. The literature on psychometrics indicates that the adaptation process is important because it provides more than just a literal translation. However, there is no technical agreement on how to conduct this process in a reliable and objective manner.^25^^,^^26^ The steps of the present study were an attempt to cover the methodological guidelines and linguistic specificities regarding both quantitative and qualitative criteria. Items 3 and 4 of the PTSD questionnaire, which contain the terms *internal* and *external reminders of trauma*, were extensively discussed and may be considered to be an example of the importance of cultural adaptation. There is no literal translation for these expressions, and suggestions made by the committee were tested on different populations before final approval was reached, in order to ensure that these items kept the same meaning as in the original version and were fully comprehensible.

The overall results showed evidence of content validity for the ITQ in its Brazilian version, since the original construct was preserved after conceptual, idiomatic, cultural and semantic issues had been carefully handled by the researchers and subsequently approved by the independent expert judges, clinical and community samples and authors of the original scale. Although the kappa statistics were not perfect, they were considered satisfactory, given that the proposal of ICD-11 regarding posttraumatic reactions is novel in the field, especially the one for complex PTSD. This may have contributed towards making the agreement between the judges only moderate.

Most studies on the ITQ have been performed using its original version or without any report of cross-cultural adaptation steps. Only in the study that reports on data from the Arabic version of the ITQ were interviews about content validity conducted with therapists.^21^ Although satisfactory qualitative evidence was found, the authors of the Arabic version suggested that the ITQ would be best administrated with the assistance of a trained professional among illiterate and poorly literate individuals. Our results did not indicate that assistance would be needed when using the ITQ in the Brazilian context, since the 35 people used to test the version did not present difficulties in comprehending any items of either questionnaire (and not even when the scores of the participants with lower education levels were evaluated separately). The aim of the ITQ is that it should be a brief self-report screening instrument for PTSD and complex PTSD, and our results indicate that this goal was achieved in the Brazilian version of the instrument.

Some important limitations of our study need to be addressed. Although we made an attempt to minimize the influence of regional speech patterns by conducting the study in three different cities, it was not possible to represent the entire Brazilian population with this sample. Also, further validation of the ITQ is needed (especially regarding construct validity) in studies with larger samples, in order to establish whether the items replicate the model of the ICD-11 properly. More robust psychometric studies are currently being conducted to achieve these goals.

These findings enable initial research using the ICD-11 model for PTSD and complex PTSD in Brazil. Future studies should focus on advancing knowledge regarding the nature, predictors, course and outcomes of these disorders in the Brazilian population. This approach is likely to contribute to the discussion of these diagnoses in an overall manner.

## CONCLUSION

The Brazilian Portuguese version of the ITQ was translated and culturally adapted to its context, and it exhibited satisfactory content validity.

## Appendix 1. Questionário de Trauma Internacional (International Trauma Questionnaire, ITQ)

Questionário de sintomas de transtorno de estresse pós-traumático

Abaixo, há uma lista de problemas e queixas que as pessoas, às vezes, têm em resposta a experiências de vida traumáticas ou estressoras. Por favor, leia cada item cuidadosamente, e circule um dos números à direita para indicar o quanto esse problema tem lhe incomodado **no último mês.**

**Table:**

	Nem um pouco	Um pouco	Moderadamente	Bastante	Extremamente
1. Ter sonhos desagradáveis que reproduzem parte da experiência ou são claramente relacionados à experiência?	0	1	2	3	4
2. Ter imagens ou memórias intensas que, às vezes, vêm a sua mente, fazendo com que você sinta que a experiência está acontecendo novamente no aqui e agora?	0	1	2	3	4
3. Evitar pistas internas da experiência (por exemplo, pensamentos, sentimentos ou sensações físicas)?	0	1	2	3	4
4. Evitar pistas externas da experiência (por exemplo, pessoas, lugares, conversas, objetos, atividades ou situações)?	0	1	2	3	4
5. Estar “hiperalerta”, vigilante ou em guarda?	0	1	2	3	4
6. Sentir-se sobressaltado ou facilmente assustado?	0	1	2	3	4
No último mês, os sintomas acima:					
7. Afetaram seus relacionamentos ou sua vida social?	0	1	2	3	4
8. Afetaram seu trabalho ou sua capacidade de trabalhar?	0	1	2	3	4
9. Afetaram qualquer outra parte importante da sua vida, como o cuidado com seus filhos, vida escolar ou acadêmica, ou outras atividades importantes?	0	1	2	3	4

Questionário de sintomas - desorganização interpessoal

Abaixo, há problemas ou sintomas que as pessoas que passaram por eventos de vida traumáticos ou estressores, às vezes, experenciam. As questões se referem a como você tipicamente se sente, como você tipicamente pensa a respeito de si mesmo, e como você tipicamente se relaciona com outras pessoas. Responda às seguintes perguntas pensando no quão verdadeira cada afirmativa é para você.

**Table:**

O quão verdadeiro isso é para você?	Nem um pouco	Um pouco	Moderadamente	Bastante	Extremamente
1. Quando estou chateado, demoro bastante tempo para me acalmar	0	1	2	3	4
2. Sinto-me anestesiado ou emocionalmente desligado	0	1	2	3	4
3. Sinto-me um fracasso	0	1	2	3	4
4. Sinto-me sem valor	0	1	2	3	4
5. Sinto-me distante ou desconectado de outras pessoas	0	1	2	3	4
6. Acho difícil ficar emocionalmente próximo de outras pessoas	0	1	2	3	4
No último mês, os problemas emocionais, as crenças sobre você mesmo e nos seus relacionamentos listados acima:					
7. Afetaram seus relacionamentos ou sua vida social?	0	1	2	3	4
8. Afetaram seu trabalho ou sua capacidade de trabalhar?	0	1	2	3	4
9. Afetaram qualquer outra parte importante da sua vida, como o cuidado com seus filhos, vida escolar ou acadêmica, ou outras atividades importantes?	0	1	2	3	4
